# Effects of Acupuncture on Breast Cancer Patients Taking Aromatase Inhibitors

**DOI:** 10.1155/2022/1164355

**Published:** 2022-09-12

**Authors:** Qing-ling Qi, Xue Han, Cheng Tang

**Affiliations:** ^1^First Teaching Hospital of Tianjin University of Traditional Chinese Medicine, National Clinical Research Center for Chinese Medicine Acupuncture and Moxibustion, 300381 Tianjin, China; ^2^School of Pharmacy, Tianjin Medical University, 300070 Tianjin, China

## Abstract

Although acupuncture has been used in clinical practice for thousands of years, it remains a controversial treatment option to help alleviate pain in cancer patients. In this study, we analyzed published material on randomized trials of acupuncture from MEDLINE published up until July 31, 2018, to assess its effects on pain experienced by cancer patients. Revman 5.0 software was used to conduct meta-analysis with pain score as the index. The results of nine randomized controlled trials involving 592 patients were analyzed and showed that acupuncture can relieve the pain caused by aromatase inhibitors. Weighted mean difference of worst pain and pain severity was -3.03, 95% CI (-3.90,-2.16) and -2.69, 95% CI (-4.08,-1.30), respectively (*P* < 0.01). This led us to conclude that acupuncture has pain relieving effects against pain caused by aromatase inhibitors.

## 1. Introduction

Aromatase inhibitors are used in the treatment of breast cancer [1]. However, these medicines can induce joint muscle pain, bone pain, urticaria, polycythemia, and other adverse reactions [2, 3]. The incidence of joint pain is related to the mechanism of aromatase inhibitors, which causes symptoms such as symmetrical arthralgia of limb joints of the wrist, knee, and fingers. Joint pain often occurs within 1 year of treatment and can reach its peak in half a year [4]. These negative side effects may cause anxiety for patients with breast cancer, thus negatively affecting their therapy.

Acupuncture is an ancient traditional Chinese medicine therapy practice, whereby needles are inserted at certain anatomical points in the body to alleviate specific symptoms. Acupuncture also involves the use of techniques such as twisting and lifting of the needle to stimulate specific parts of the human body to treat diseases [5, 6].

However, acupuncture is a controversial therapy for the chronic pain, as it cannot be proven by biomedicine. Traditional acupuncture theory is not based on anatomical structures but rather landmarks such as meridians and nonphysiological processes. Therefore, it was reported that acupuncture has short-term physiological effects, but there is no information explaining what these effects are and the impact on the patients' therapy. Due to the lack of data, many clinical practitioners do not accept this concept as a treatment option.

In this study, we perform a data meta-analysis of high-quality trials of acupuncture for the therapy of the pain caused by aromatase inhibitors. These results could be used as evidence for acupuncture's ability to relieve pain caused by aromatase inhibitors.

## 2. Method

### 2.1. Patients

The protocol of the analysis was as follows: literature that was published from 2007 to 2018 was selected. Studies were considered eligible for this present study if they met the following conditions: (1) the patients had been on aromatase inhibitor treatment and received acupuncture needling therapy; (2) the patients received either sham acupuncture or no acupuncture (control group); (3) the primary endpoint was measured more than 4 weeks after the therapy of acupuncture; (4) all of the patients were randomly divided into groups. Cases were selected for our meta-analysis if they met the following conditions: (1) that the patient had received aromatase inhibitor therapy for the treatment of breast cancer. (2) The patient was willing to receive acupuncture treatment for treatment of pain associated with the use of aromatase inhibitors. The patients were then randomly divided into two groups. One of the groups would receive acupuncture needling therapy, and the other group would be subjected to placebo treatment. The patients were then assessed 4 weeks after acupuncture therapy, and results were recorded.

The results for each study were analyzed by the authors of this manuscript. The effect size was entered into a meta-analysis program using the RavMan (version5.0) software. Both fixed effects and random effects were evaluated.

### 2.2. Data Analysis

RavMan (version5.0) software was used for meta-analysis. Adopt first *χ*^2^ test which was used to analyze the heterogeneity of the study, and the standard was *α* = 0.05; when the *P* value > 0.05, it indicates that the quality of each study is homogeneous, and the fixed effect model was selected; otherwise, random effects model was selected. Odds ratio was used for counting data and the measured data was expressed by weighted mean difference. Both of them were based on 95% confidence interval as the effect comprehensive scale.

## 3. Results

According to the search and data collection methods, nine studies met the inclusion criteria, which included a total of 592 patients (Table 1). The nine trials included random grouping with one group set up to receive placebo acupuncture. There are only 4 items mentioned in the random grouping method. The method used for acupuncture was not mentioned for most of the cases. The inverted funnel model was drawn according to the score of pain, and the results showed the scattered data were unevenly distributed on the left and right, suggesting that there may be a certain bias.

Evaluation of analgesic effect: nine studies show *χ*^2^ = 5.32, *P* > 0.05, indicating no statistical heterogeneity. The results of fixed effect model showed that the difference between the groups before and after acupuncture was statistically significant (*P* < 0.01), indicating that acupuncture had a better inhibitory effect on relieving the pain caused by aromatase inhibitor.

**Figure 1:**
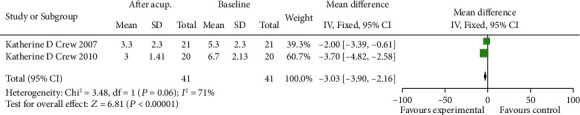


**Figure 2:**
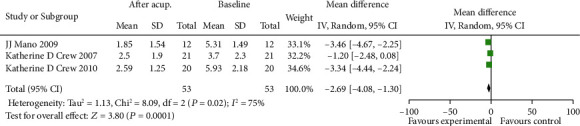


To further determine the effects of acupuncture, the pain scores that were recorded before and after the therapy were evaluated by using the following indicators: worst pain, WOMAC-pain, and pain severity. The results showed that there was statistical heterogeneity in the scores before and after the therapy of acupuncture. Weighted mean difference of worst pain and pain severity is -3.03, 95% CI (-3.90,-2.16) and -2.69, 95% CI (-4.08,-1.30), respectively (Figures 1 and 2). The results showed that the difference between the two groups was statistically significant (*P* < 0.01).

Antifatigue effect and influence on mood improvement: research showed that acupuncture can also improve fatigue symptom of patients. After 6 weeks of acupuncture, fatigue decreased significantly in patients. Acupuncture also had a greater impact on patients' emotions. After treatment, patients' mood showed some improvement.

Based on the meta-analysis, results showed that acupuncture could improve the quality of life of breast cancer patients after the treatment by using aromatase inhibitors.

## 4. Discussion and Conclusion

At present, the mechanism of acupuncture anesthesia and analgesia has been explored [16, 17]. The proven mechanism include three parts. One of them is the neurophysiological mechanism including the peripheral afferent pathway of acupuncture signals, the central transmission pathway of acupuncture signals, the integration of acupuncture signals, the pain signals in the central nervous system, and the activation of some related pain modulation structures in the brain; the other part is the neurochemical mechanisms including *β*-endorphin, enkephalin, and dynorphin; the last part is the molecular mechanism, such as electroacupuncture can regulate the release of TNF-*α*, interleukin-1*β*, interleukin-6, and prostaglandin-E2. They could immediate the long-term analgesia. The quality of research methodology and nonstandard reporting could affect the quality of clinical research [18, 19]. We suggest that clinical acupuncture research design should be standardized [20]. There are many meta-analyses on acupuncture-assisted treatment of breast cancer, but none of them mention the improvement of symptoms such as pain in breast cancer patients after the therapy of aromatase inhibitor. There is no reasonable analysis of clinical heterogeneity analysis, which has limited value for clinical guidance. This study summarized the latest trail information available on the effects of acupuncture for symptoms of breast cancer patients after aromatase inhibitor treatment. And we carried out a reasonable clinical heterogeneity and subgroup analysis. In addition, the different acupoints and treatment methods selected in the literature included in the study may impact the results of the therapy. Therefore, whether acupuncture can improve the quality of life of patients with breast cancer needs to be further verified by high-quality, large-sample, randomized controlled studies.

## Figures and Tables

**Table 1:** Patient clinical characteristics.

Name of the author	Median age	Acupuncture	Sham acupuncture	Waitlist control
T Bao [7]	61	23	24	—
J.J. Mao [8]	60	22	22	23
T Bao [9]	61	23	24	—
J J. Mao [10]	59	22	22	23
J Bauml [11]	59	22	22	23
JJ Mao [12]	59	12	—	—
KD Crew [13]	59	21	—	—
K D. Crew [14]	57	20	18	—
DL Hershman [15]	60	110	59	57

## Data Availability

The data used to support the findings of this study are included within the article.
